# Nanometal Skin of Plasmonic Heterostructures for Highly Efficient Near-Field Scattering Probes

**DOI:** 10.1038/srep31113

**Published:** 2016-08-09

**Authors:** Gianluigi Zito, Giulia Rusciano, Antonio Vecchione, Giuseppe Pesce, Rocco Di Girolamo, Anna Malafronte, Antonio Sasso

**Affiliations:** 1 University of Naples Federico II, Dept. of Physics E. Pancini, via Cintia 80126-I, Naples, Italy; 2 Istituto Nazionale di Ottica (INO) Consiglio Nazionale delle Ricerche, via Campi Flegrei 34- 80078 Pozzuoli, Italy; 3 Istituto SPIN U.O.S. Consiglio Nazionale delle Ricerche, via Giovanni Paolo II 132, 84084, Fisciano, Italy; 4 University of Salerno, Dept. of Physics, via Giovanni Paolo II 132, 84084, Fisciano, Italy; 5 University of Naples Federico II, Dept. of Chemical Sciences, via Cintia 80126-I, Napoli, Italy

## Abstract

In this work, atomic force microscopy probes are functionalized by virtue of self-assembling monolayers of block copolymer (BCP) micelles loaded either with clusters of silver nanoparticles or bimetallic heterostructures consisting of mixed species of silver and gold nanoparticles. The resulting self-organized patterns allow coating the tips with a sort of nanometal skin made of geometrically confined nanoislands. This approach favors the reproducible engineering and tuning of the plasmonic properties of the resulting structured tip by varying the nanometal loading of the micelles. The newly conceived tips are applied for experiments of tip-enhanced Raman scattering (TERS) spectroscopy and scattering-type scanning near-field optical microscopy (s-SNOM). TERS and s-SNOM probe characterizations on several standard Raman analytes and patterned nanostructures demonstrate excellent enhancement factor with the possibility of fast scanning and spatial resolution <12 nm. In fact, each metal nanoisland consists of a multiscale heterostructure that favors large scattering and near-field amplification. Then, we verify the tips to allow challenging nongap-TER spectroscopy on thick biosamples. Our approach introduces a synergistic chemical functionalization of the tips for versatile inclusion and delivery of plasmonic nanoparticles at the tip apex, which may promote the tuning of the plasmonic properties, a large enhancement, and the possibility of adding new degrees of freedom for tip functionalization.

Tip-enhanced Raman scattering (TERS) spectroscopy allows the detection of molecular Raman spectra with near-field resolution, thus pushing vibrational identification of biomolecular distributions to the extreme nanoscale^1^,^2^,^3^,^4^,^5^. Light scattering is generated by the local electromagnetic field resonating into a metal structured tip acting as a nanosource and nanoantenna for reirradiating evanescent light in the far field^6^,^7^,^8^,^9^,^10^,^11^,^12^,^13^,^14^,^15^. Similarly, scattering-type scanning near-field optical microscopy (s-SNOM) enables exploring the optical properties of materials by elastic photon scattering^16^,^17^,^18^,^19^,^20^,^21^,^22^ or absorption^23^,^24^. Both nanospectroscopies provide fundamental keys for looking into materials at molecular scale.

While scanning tunneling microscopy tips used for TERS consist typically of etched bulky silver or gold probes with a sharp apex^2^, TERS probes based on atomic force microscopy (AFM) cantilevers are typically fabricated by physical vapor depositions of metal coatings on commercial silicon probes^4^,^25^,^26^. For tips made of a continuous plasmonic medium - either solid or with a smooth coating^6^ - near field enhancement relies on a combination of lightning rod-type effects and surface-plasmon excitations. The near field at the tip apex results to be significantly amplified for longitudinally polarized far field excitation^27^. Moreover, when the tip is close to an additional bottom plasmonic mirror, a much higher gain is achieved in the gap between tip and bottom substrate. In this configuration of gap-mode TERS, further enhancement of ~10^2^ can be obtained, very useful for most of biomolecular applications^11^,^12^,^25^, although limiting TER spectroscopy to thin molecular layers.

Vapor deposition typically produces a monolayer of NPs resulting from the dewetting of the metal on the probe surface, which gives rise to discontinuous random nanoislands or to a continuous, rough film^28^. Other fabrication schemes have emerged such as isolated NP deposition^29^,^30^, electrochemical^31^,^32^ and photoreduction-induced NPs growth^33^, gold film coating by template stripping^6^, or pulsed electrodeposition^34^. Although many efforts demonstrate the importance of engineering the plasmonic response of TERS probes^3^,^4^,^12^,^28^,^35^,^36^,^37^, tuning the response of NPs is still a challenge. In addition, properties like spatial resolution and enhancement factor of the probe depend dramatically on the apical geometry^35^. In particular, lack of control in the plasmonic coupling between closely spaced metal random nanoislands or nanoparticles randomly evaporated on the probe usually do not allow adequate control of the generated optical response of the apex.

In this work, we explore new strategies of functionalization of near-field scanning probes offered by NPs inclusions into block-copolymer (BCP) micelles^38^,^39^. The metal NPs are, in this case, first synthesized and then deposited on the probe in order to introduce a certain control of their geometry and thus of the plasmonic properties. Recently, we have verified that clusters of AuNPs of 15 nm on AFM tips may allow nongap-TER spectroscopy of thick biosamples^40^. Yet, to date a systematic study of the properties of closely-spaced clusters of small NPs for TERS applications has not been carried out. Analogously, SERS nanostructures fabricated by loading NPs into BCP micelles have revealed excellent properties in terms of tunability, versatility and near-field optical properties^41^,^42^. However, to the best of our knowledge, implementation of metal-BCP nanocomposites for scanning probes coating has not been reported yet. Here, we coat AFM scanning probes *via* self-assembling monolayers (SAMs) of BCP micelles loaded either with clusters of silver nanoparticles (AgNPs) or bimetallic structures of mixed species of silver and gold nanoparticles (AuNPs). In particular, we will refer to this bimetallic architecture as Ag@AuNP for the reason clarified later. Firstly, SAMs of AgNPs-loaded micelles allow a continuous coating of the tips with a sort of nanometal skin. The monolayer consists of geometrically confined nanoislands made of aggregates of NPs separated by the polymer shell. Unlike other colloidal synthesis and growth processes, BCP micelles limit the size dispersion of the closely-spaced NPs nucleated inside them. In addition, the micelles constitute a template that controls the reproducible patterning of the metal seeds and also preserve the NPs from ambient air interaction and oxidation. Secondly, we investigate the possibility of combining silver and gold NPs into BCP micelles for both tuning of the plasmonic response and having a more biofriendly contact layer offered by the AuNPs at the tip apex. The multiscale fractal aggregation of NPs favors a large enhancement factor^41^,^42^,^43^ induced by their mutual interaction. A thorough characterization of the nanostructured tips is carried out on several standard Raman analytes. This analysis demonstrates the tips to be capable of an excellent enhancement factor and spatial resolution. In particular, in a gap-mode TERS experiment we provide, for the first time, the bi-analyte tip-enhanced Raman scattering (BiA-TERS) proof of single molecule detection with nanoscale spatial resolution. In addition, the amplification expected on the outer surface of the apex clusters can be large-enough to be exploited for nongap-TERS experiments as well, which can be useful for nanospectroscopic investigation of thick biosamples. In fact, to this purpose, our TERS tips are tested on *Bacillus subtilis* spores in backscattering configuration (epi-illumination).

Our approach introduces a synergistic chemical functionalization of the tips for versatile inclusion and delivery of plasmonic nanoparticles at the tip apex, which may promote the tuning of the plasmonic properties, a large enhancement, and the possibility of adding new degrees of freedom for tip functionalization.

## Results

### Tip fabrication: synthesis

**Figure 1:**
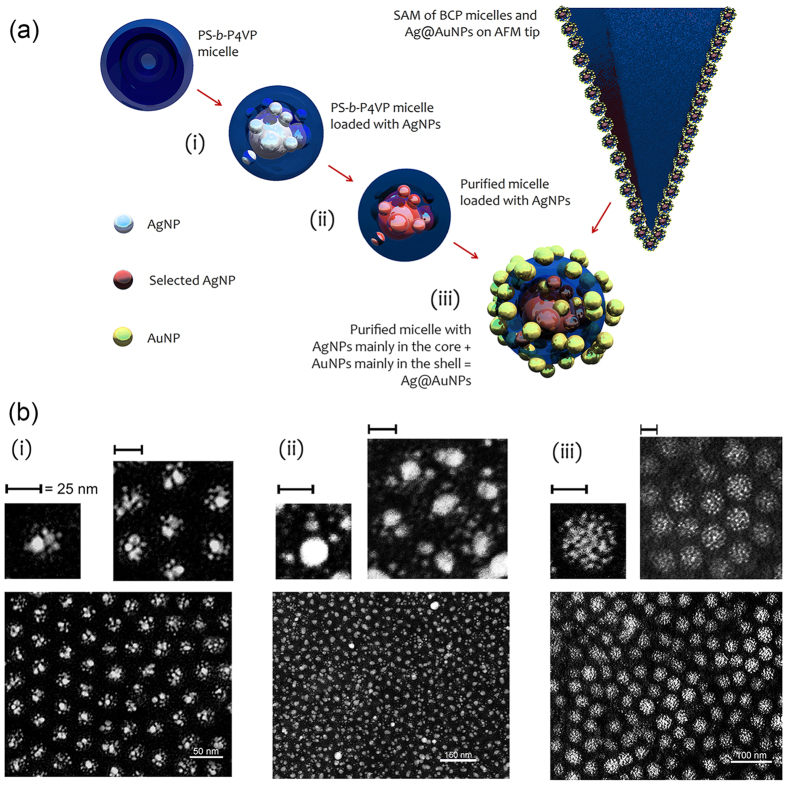
Schematic process of SAM coating of Si-AFM tip by BCP micelles loaded with AgNPs and Ag@AuNPs. (**a**) In step (i), PS-*b*-P4VP micelles are loaded with AgNPs, mainly in the core of P4VP copolymer^42^. (ii) Next, overgrowth of AgNPs in presence of an excess of Ag^+^ in solution, produces bigger NPs; Ag-BCP nanocomposites are purified by density gradient centrifugation to select micelles with larger NPs in the core. In the next step (iii), Au precursor is added to the solution of micelles preloaded with AgNPs; AuNPs, formed with excess of NaBH_4_, were evident in the BCP micelles by EDX analysis (Fig. S1) and UV-vis spectroscopy (Fig. 2), and morphologically evident in the PS shell at TEM inspection reported in panel (b). Therefore, we tentatively describe this structure as consisting of AgNPs mainly in the P4VP core and AuNPs mainly in the PS shell (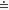 as Ag@AuNPs). Finally, both solutions of BCP with AgNPs or Ag@AuNPs are used to coat Si-AFM tips by dip coating. (**b**) TEM micrographs (inverted colormap) of the three steps of the process depicted in panel (a). In particular, the modification of the size of the NPs, evident from the comparison of the consecutive SAMs spin-coated on glass, pointed out the formation of AgNPs of 15–20 nm surrounded by smaller Ag seed satellites in the P4VP core (ii). An outer shell that we ascribe to tiny AuNPs (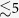 nm) appears in (iii) as described in the main text. Top insets are magnified regions of the corresponding bottom scans. Scalebars are 50, 150 and 100 nm in (i), (ii) and (iii), respectively, whereas scalebar = 25 nm in all top insets.

In Fig. 1(a), a scheme of the coating procedure is shown. Synthesis of nanoparticles and purification procedure is depicted as a multiple-step process. The three main steps are labeled as (i–iii). In Fig. 1(b), morphological characterizations are labeled accordingly. Poly(styrene-*block*-4-vinylpyridine) (PS-*b*-P4VP) block copolymer was used to form inverse micelles, with core of P4VP and shell of PS, by dissolving the diblock copolymer (19,200-*b*-10,400, Polymer Source, Inc.) into a solution of toluene/THF according to our previous recipe^41^. Micelles had an average diameter of ~36 nm. (i) *In situ* reduction of metal ions Ag^+^ (from AgNO_3_), protonated with micellar core pyridine, produced aggregates of metal nanocrystals nucleated within the P4VP core of ≃26 nm. Reduction to metal was accomplished by an excess of NaBH_4_. (ii) So-obtained solutions were purified by centrifugation and filtration in an iterative fashion by a density gradient procedure^44^ in order to eliminate unreacted salts and select for deposition only heavier micelles (Methods). Typically, NPs filtered by purified micelles were characterized by a bigger nanoparticle core of 15–20 nm with residual satellite seeds of size from 1 to 5 nm. Bigger NPs were the result of prolonged growth in excess of Ag^+^ in solution. (iii) Next, further addition of Au^3+^ from HAuCl_4_ ⋅ 3H_2_O to the solution of micelles, preloaded with already formed AgNPs in the core, gave rise to an additional inclusion of gold nanoparticles 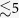 nm in the BCP micelles (Methods). We will refer to this bimetallic heterostructure as Ag@AuNPs nanoisland. In Fig. 1(b), the SAM patterns of (i) AgNPs, (ii) overgrown purified AgNPs and (iii) Au@AgNPs were obtained by spin-coating the solutions on glass for TEM inspection (Methods), and correspond to the above mentioned three steps. In particular, the presence of AuNPs embedded in the BCP micelles was indicated by two concurrent inspections, the morphology correlated to the energy dispersive X-ray characterization, and the UV-vis optical characterization of same films. Firstly, we observed a modification of the initial nanoisland diameter from 26 nm (on average) in panel (i) (AgNPs preloaded and present only in the core) to 37 nm in panel (iii) (additional AuNPs). Same starting solution was used for all the three steps reported in Fig. 1(b). The presence of mixed species of Ag- and Au-NPs in the heterostructures was confirmed by energy dispersive X-ray spectroscopy analysis as a function of the molar ratio between Ag and Au (Methods), shown in Fig. S1 in Supplementary Information (SI). Therefore, since P4VP cores were prefilled with AgNPs and given the high affinity of gold toward PS^45^, additional AuNPs were likely embedded mainly in the PS shell. However, AuNPs presence in the core was not excluded. In either cases, mixing silver and gold NPs broadens the plasmonic response offered by the system, as discussed below.

**Figure 2:**
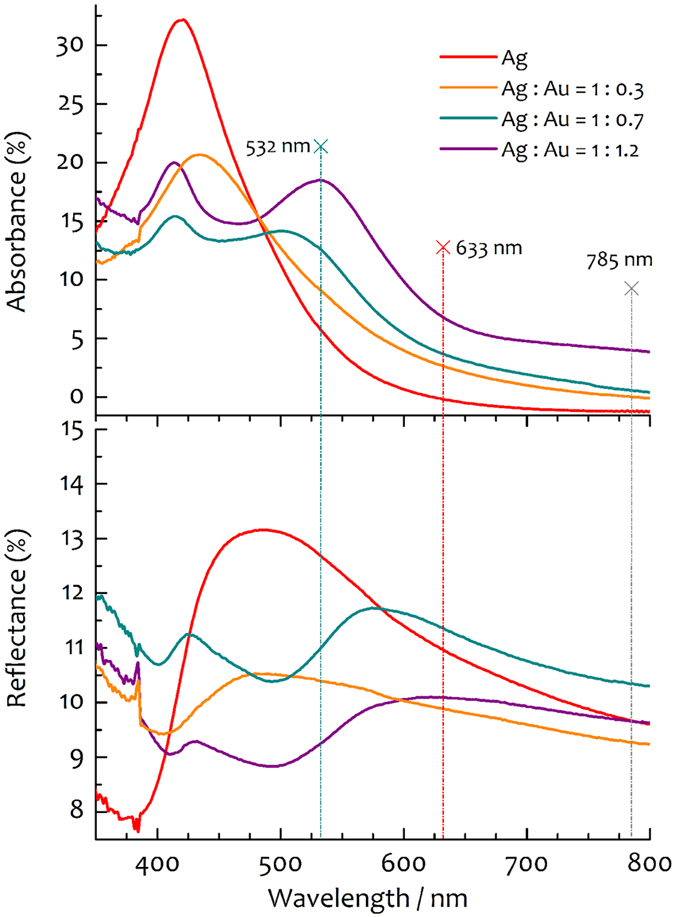
UV-vis characterization. Absorbance and reflectance spectra of nanoislands obtained from templates of BCP micelles loaded with clusters of AgNPs and bimetallic Ag@AuNPs with amount ratios Ag: Au as indicated in the legend (from EDX, Fig. S1). UV-vis curves were acquired in transmission (extinction) and reflection (scattering) on films spin-coated on glass coverslips, hence determining the absorbance contribution. For AgNPs, a large scattering contribution is measured and peaked at 500 nm against absorption peaked at 420 nm (red lines). For Ag@AuNPs, there is a significant variation in both absorption and scattering coefficients. The main scattering band, initially peaked at 480 nm (yellow line), progressively moves to 575 nm (cyan line) and then to ~630 nm (violet line) extending in the infrared. These bands are ascribed to additional clustering of AuNPs mixed with AgNP aggregates.

Figure 2 shows the UV-vis spectra obtained from films deposited on glass. We can see a strong scattering contribution (reflectance) from AgNP clusters at ~500 nm, consequence of the strong local field produced by NP interaction, which is also expected to balance in part the lower radiative efficiency of isolated NPs 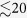 nm^46^. The redshift and broadening of the main scattering band is ascribed to the random aggregation of the polydispersed nanoparticles forming the clusters^43^,^47^ and to inter-cluster coupling influence^42^. Templates of BCP micelles totally loaded with AuNPs were also fabricated for comparison. However, they were characterized by small Au seeds showing much larger absorption peaked at 540 nm, and were not used for TERS-active coating at this stage. Varying the molar ratio of AgNO_3_ and HAuCl_4_ ⋅ 3H_2_O in the preparation of the solution (so the relative atomic concentration of Ag and Au, respectively 1 : 0.3, 1 : 0.7 and 1 : 1.2, Methods) gave rise to the emergence of scattering peaks from 480 nm to 575 nm, with a pronounced contribution also at 633 nm and a tail up to the infrared (Fig. 2). The redshift of the lower energy peak evident in the absorbance spectra for mixed Ag- and Au-NPs was mainly ascribed to the hybridization between closely spaced NPs. In Supplementary Fig. S2, we show the scattering cross section spectrum calculated considering a cluster of mixed AgNPs (mainly in the core) and AuNPs (mainly in the shell) arranged into a nearly spherical aggregate. The main scattering bands so determined and arising at ~400–460 and 550–570 nm are qualitatively consistent with the experimental ones.

Combining silver and gold for NPs synthesis in BCP micelles was explored for tuning the plasmonic response for visible excitation, but also for providing a biofriendly contact coating on the tip. While silver possesses superior scattering efficiency than gold at 532 nm, it is also prone to oxidation and chemical interaction with the explored environment. On the other hand, gold is more chemically inert but produces larger heat transfer for nanoparticles of the order of 10 nm. Therefore, as a proof-of-principle experiment, we combined Ag and Au aiming at achieving both high scattering efficiency and chemical stability using a silver core to boost the local field amplification; and limiting the excitation intensity to ~10^5^ W/cm^2^ in TERS measurements.

### Tip fabrication: dip-coating

**Figure 3:**
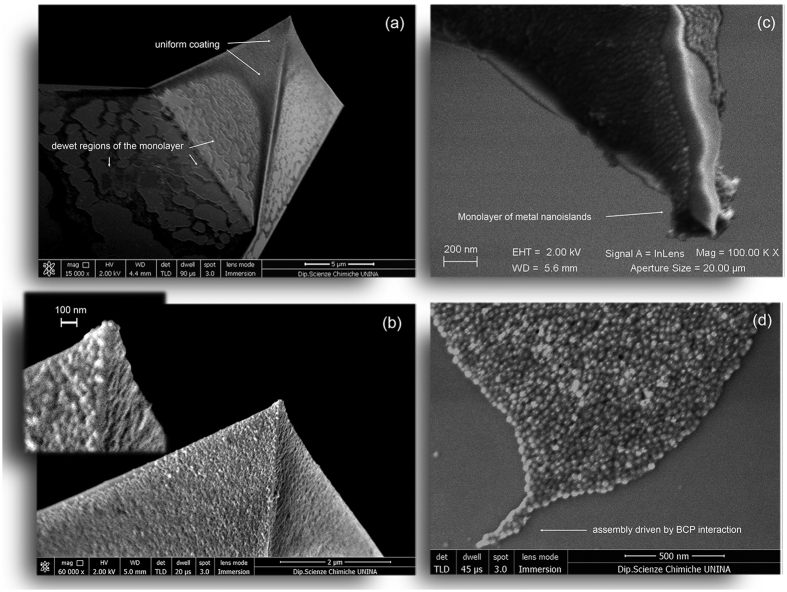
SEM characterization of Si-AFM probes coated with SAMs of metal nanoislands. (**a**) SEM micrograph of an Arrow^©^-type AFM probe coated with clusters of AgNPs showing a uniform monolayer on the pyramidal tip with dewet regions at the base. (**b**) Magnified version of (**a**) with inset showing a region close to the apex where contrast is increased to resolve the structure of close-packed nanoislands. (**c**) Arrow^©^-type tip coated with Ag@AuNP nanoislands: the ripped coating reveals a monolayer structure of metal nanoislands, which resembles a sort of nanometal skin on the probe. (**d**) Region of residual coating present on the base shaft close to the tip, where it is possible to appreciate the close-packed assembly produced by attractive forces during solvent evaporation. These are capable of producing granular patterns commensurate with the geometry of the tip apex. Please note, as for instance, the stinger-shaped assembly indicated by the arrow. Such a kind of terminations appear also on the apex of (**b**).

The BCP provided the network for building controlled assemblies of nanoislands commensurate with the apex geometry of etched sharp probes, therefore enabling coverage of the tip. The solvent evaporation produces nearly periodic SAMs as a delicate balance among electrostatic and steric repulsion among micelles and attractive capillary forces in the solvent^48^. In case of a 3*D* geometry like a pyramidal tip with a sharp apex, an accurate control of the deposition geometry was necessary. In order to enable the tip apex coverage, it was crucial to extract the tip from the liquid with the apex pointing toward the liquid/air interface. To this end, the BCP-metal solution in toluene/THF was bubbled from the free surface of a micropipette dispenser in a controllable and steady fashion by tuning the expelled volume of liquid. Then, the angle between the cantilever shaft and the vertical was adjusted to achieve the best coating. Only the cantilever was immersed in the bubble and monitored in real time with a custom optical microscope during the extraction. SAMs produced under optimal dip-coating angle allowed forming a coating of close-packed BCP micelles on the tip. Hence, nearly spherical nanoislands of AgNPs or Ag@AuNPs, with gap between nanoislands even below 5 nm, were obtained on the AFM tips. Experimental results are shown in Fig. 3, where representative SEM micrographs of Si-AFM probes coated with metal nanoislands are shown. In particular, we used Arrow^©^-type probes (NanoWorld, Switzerland) and TESPA^©^-type AFM tips (Bruker) in this work. In Fig. 3, we show two Arrow^©^-type tips coated with AgNP and Ag@AuNP nanoislands, respectively. From the visibly ripped coating in panel (c), it is clearly possible to see a perfect monolayer structure of metal nanoislands, which resembles a sort of nanometal skin on the probe. A detailed region of this monolayer is also shown for better clarity in panel (d), acquired from the base shaft. In particular, in Fig. 3(d), it is possible to appreciate the close-packed assembly produced by attractive forces during solvent evaporation that shrink the metal structure. Thus, the BCP interaction is capable of producing granular patterns commensurate with the geometry of the tip apex, as can be seen in the low-left corner from the stinger-shaped assembly terminating with a single nanoisland.

The BCP component, useful for protecting the tip from silver oxidation before use, was removed immediately prior to the measurements by UV exposure at 254 nm with a commercial lamp (24 h of exposure). Unavoidable polymer residue contamination gave rise to a blank TERS signal consisting of a carbonaceous background at 1300–1550 cm^−1^. This was one order of magnitude lower than the silicon Raman band at 518 cm^−1^ from the tip. Although at the cost of introducing an undesirable background signal (of limited intensity), the polymer residues were expected to increase the immobilization of the clusters, also favoring a 3*D* arrangement mediated by the BCP linkers.

Importantly, the BCP templates the nanoisland arrangement so to produce highly interacting granular structures of NPs on the tip, nanometrically distanced by the micelle shells^41^. In a recent related study, Taguchi *et al*.^49^ concluded that discrete arrangements of disconnected NPs on the tip provide a larger TERS enhancement factor. Our study supports their conclusion. Moreover, by adding a further degree of freedom to the complexity of the plasmonic architecture, our approach moves the focus on the multiscale enhancement of disconnected clusters nucleated into micelles and hierarchically patterned into nanoislands.

### Theoretical background and motivation

The fractal, multiscale heterogeneity in the architecture of the nanoisland is intended to favor large enhancement factors in the gaps of concatenated NPs^50^,^51^. Increasing the number of clustered NPs has been associated to larger SERS enhancement factors^42^,^43^,^47^. Moreover, symmetry breaking in heterodimers^52^,^53^ and more complex configurations of nanoparticles^50^,^51^ provides strong interaction between plasmon modes. Among other benefits such as cascade amplification^50^,^51^, their complex plasmon hybridization enables enhanced coupling with the far-field radiation^52^ and large enhancement also in more simple geometries of mismatched NPs^54^. Concurrently, the heterostructures are confined within BCP micelles with limited polydispersity, therefore their overall size is still compatible with the apex size of the tip. This design was prompted by our previous experiments with surface-enhanced Raman scattering (SERS) substrates made with the planar version of the same BCP-metal patterns of AgNPs, which revealed excellent SERS enhancement and reproducibility as favored by the confinement and growth of the NPs inside the BCP micelles and pattern uniformity^41^.

The approximated morphology of the typical clusters shown in Figs 1(b) and 3 was used as a model for numerical simulations. Previous AFM inspections of the BCP pattern morphology spin-coated on Si wafer gave further indications on the geometry of the cluster^41^,^48^. Finite element method (FEM) simulations were carried out solving the full retarded electrodynamic problem (Methods). Limited by technical constraints, our simulations were focused on repre.sentative apical structures consisting of a single cluster of Ag@AuNPs onto a Si tip. While the inherent distribution of silver and gold in the cluster may change the plasmon resonances landscape of the system, the basic principles that we want to highlight depend only on the presence of aggregates of NPs at the tip. Hence, the conclusions are the same for pure AgNPs or hybrid Ag- and AuNPs clusters. Two examples are here illustrated to sketch the main characteristics found. These examples aim at supporting the idea that confined clusters of NPs may be a good choice for TER spectroscopy applications.

Spherical nanoparticle geometries are used as a first approximation. The 3*D* assembly aims at simulating NPs linked by polymer. The gaps between the various NPs is intentionally kept above 0.5 nm, since several reports indicate that for gaps <0.4 nm quantum effects become relevant and near-field enhancement is quenched by tunnelling^55^,^56^,^57^. Therefore, the level of enhancement here reported is a conservative estimate of what can be achieved with smaller gaps.

**Figure 4:**
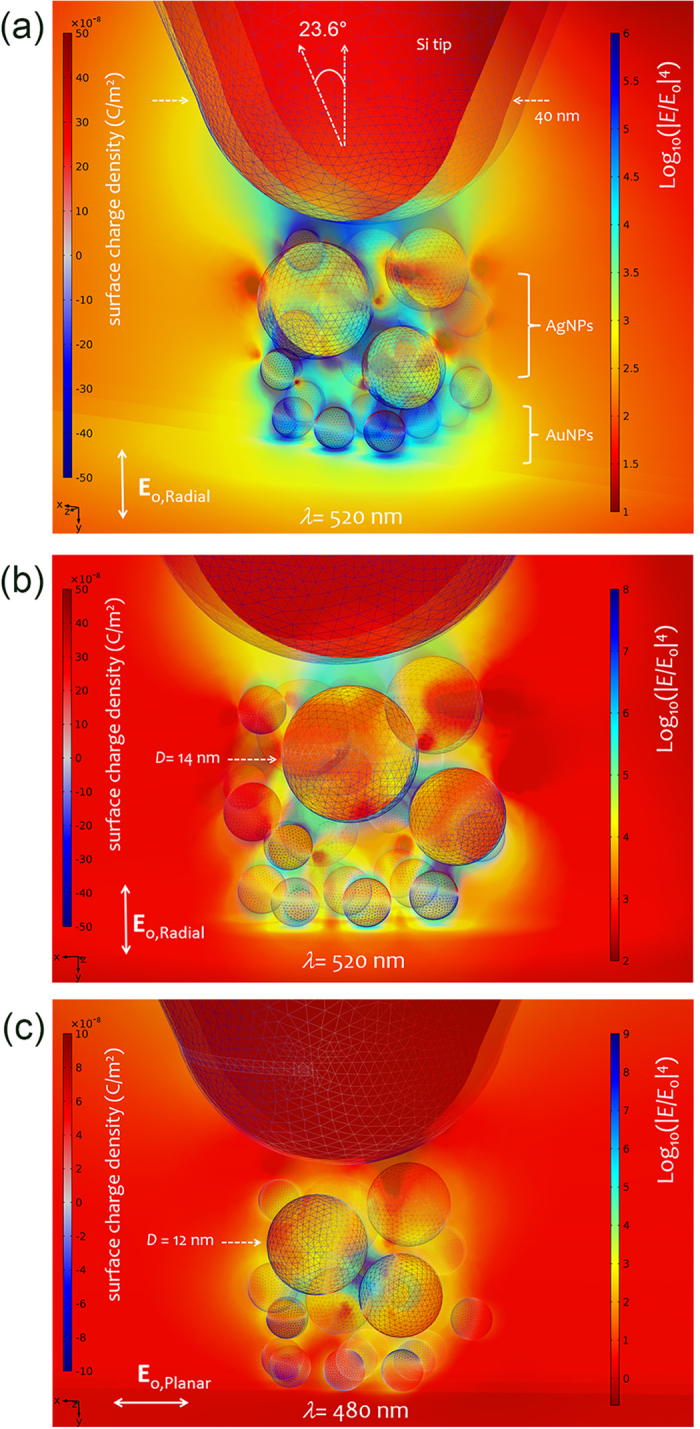
Numerical simulations of representative apical clusters at the tip apex of our TERS probes. (**a**) Enhancement factor spatial distribution approximated as the four power of the norm *E* of the scattered electric field normalized to the incident radially polarized wave field, of amplitude *E*_o_ at *λ* = 520 nm (bottom illumination); surrounding medium index *n* = 1.4. The tomographic representation of the field amplification is rendered by means of 3 overlaid transparent cut planes at *z* = −7, 0, 8 nm and a *y*-planar cut 0.5 nm below AuNPs. Colormap on the left represents the surface charge density calculated on wireframed nanoparticles and tip. The enhancement factor (logscale) on the right is saturated at 10^6^: regions of such level fill and surround the apical cluster. (**b**) Same as in (**a**), with nonsaturated colormap of enhancement factor and different angle of view. Please note the chain coupling from the center to the peripherical NPs that transmits local field amplification to the surface of AuNPs. (**c**) Enhancement factor distribution as in (**a**), but for a slightly different configuration of NPs (the bigger is 12 nm) for excitation at 480 nm, with plane wave polarization along the *x*-axis. See text for details.

In Fig. 4(a), an example of the electromagnetic distribution produced by a 3*D* cluster is shown. The enhancement factor is approximated as the fourth power of the local field gain, |*E*/*E*_o_|^4^ (Methods). The representative structure consists of a Si tip with radius of curvature ≃20 nm and a cluster of AgNPs (bigger particle of 14 nm) with a bottom layer of AuNPs. The Si tip has a length of 450 nm and is enclosed into a perfectly absorbing medium that makes it virtually infinite for what concerns backreflections. As dielectric environment, an effective biofilm medium having refractive index *n* = 1.4 was considered. A radially polarized field with longitudinal component *E*_o_ was set as incident radiation. This simulates our experimental linearly polarized laser partly converted by a liquid crystal plate into a radially polarized beam. This last is focused on the tip through an inverted objective with N.A. = 0.8.

In Fig. 4(b), the same geometry of Fig. 4(a) is reproduced with a slightly different angle of view. In Fig. 4(c), a slightly modified geometry is considered. In this case, a linearly polarized plane wave along the *x*-axis is taken into account because typically present in the experiments. Then, the freespace wavelength *λ* is scanned over a broad spectral range to outline the landscape of localized surface plasmon resonances.

Let us summarize the main results of interest to our discussion. The heterostructure provides a rich variety of localized surface plasmon resonances. The hybrid plasmon modes among nanoparticles give rise to resonances that span all the visible range, also redshifted towards lower energies depending of the sizes of the NPs and gaps (here limited to 1 nm). In the example of Fig. 4(a), the highest amplification at *λ* = 520 nm, up to 10^8^, is produced in the resonant geometry provided by the bigger NPs in the center. In general, we observe for these randomly arranged NPs that gap sizes between NPs rule the maximum value of achievable enhancement factor. Figure 4(c) shows a slightly varied geometry consisting of a smaller NP in the core with a minimum gap of 0.5 nm (still, with good approximation, within the classical model validity^56^). This provides a resonant structure at 480 nm, for planar polarization, and an increase of the hottest hot-spot gain from 10^8^ to 10^9^. Larger enhancements can also be found in more favorable geometries with gaps ~1 nm, depending on the nanoparticle concatenation geometry^54^.

A relevant point to be noted is that the complex hybridization between all NPs may transfer the enhancement also to the cluster’s outer surface through concatenated chains of NPs. As shown in Fig. 4(b), the maximum enhancement factor is reached in the gap close to the smaller NP of the misaligned chain on the right. On the contrary, in Fig. 4(c), although the hot-spot has a larger local field (10^9^), the amplification at the AuNP layer diminishes because the beneficial coupling among concatenated NPs is lost along the *x*-axis. In all these simulations, we have used AuNPs in the outer part of the cluster. However, considering a more heterogeneous distribution of NPs (or only AgNPs) does not invalidate the basic mechanism here underpinned. The attenuation of the amplification found when passing from the inner gaps to the outer surface (where molecules are expected to be probed) is typically of 3 orders of magnitude. Please see, *e.g.*, the cut plane 0.5 nm below the AuNP surfaces in Fig. 4(a,b) where enhancement factors of 10^5^–10^6^ can be achieved. Despite the attenuation, these values are still higher than what theoretically expected for solid metal tips in nongap-TERS condition^58^. Therefore, suitable clusters-coated tips may be advantageous for TER spectroscopy on thick biosamples for which a bottom plasmonic substrate cannot be used^40^. It is worth mentioning that the values predicted for the enhancement factor of such a kind on NP aggregates are in good agreement with our previous experimental characterizations conducted with planar surface-enhanced Raman scattering (SERS) substrates of AgNPs^41^.

In addition, we speculate that even better results could be achieved since chains on NPs may give rise to gap hot-spots as large as 10^11^ with outer surface enhancement of 10^9^ 50,54. In this case, it would be possible to reach the largest amplification of 10^9^ of gap-mode TERS^58^ without the need of a bottom plasmonic mirror.

We considered NPs with minimum diameter of 5 nm. Size effects on the plasmon resonance due to quantum electron confinement have been demonstrated for NPs <10 nm^59^. Actually, more relevant deviations from Mie theory occur for NPs <5 nm. Since we were mainly interested to plasmon coupling effects in concatenated NPs, we disregarded any quantum size effect in a first approximation.

### Experimental Application: TERS

#### Experiments on SWCNTs

In this work, we will focus on AFM probes coated with AgNPs and Ag@AuNPs with ratio 1 : 0.7 (Fig. 2), for which we found the best results at our excitation wavelength, probably because of their larger scattering efficiency. We applied AgNP and Ag@AuNP coated near-field probes for several TERS experiments. The experimental setup is described in Methods. Firstly, we used single-walled carbon nanotubes (SWCNTs) to determine optical contrasts and spatial resolutions of the TERS probes. For these experiments, excitation laser wavelength was at 532 nm. SWCNTs were spin-coated on a commercial glass coverslip. We used TESPA tips (Bruker) with nominal radius of curvature of 8 nm, in intermittent contact mode (300 kHz), as supporting AFM probes coated with Ag clusters.

**Figure 5:**
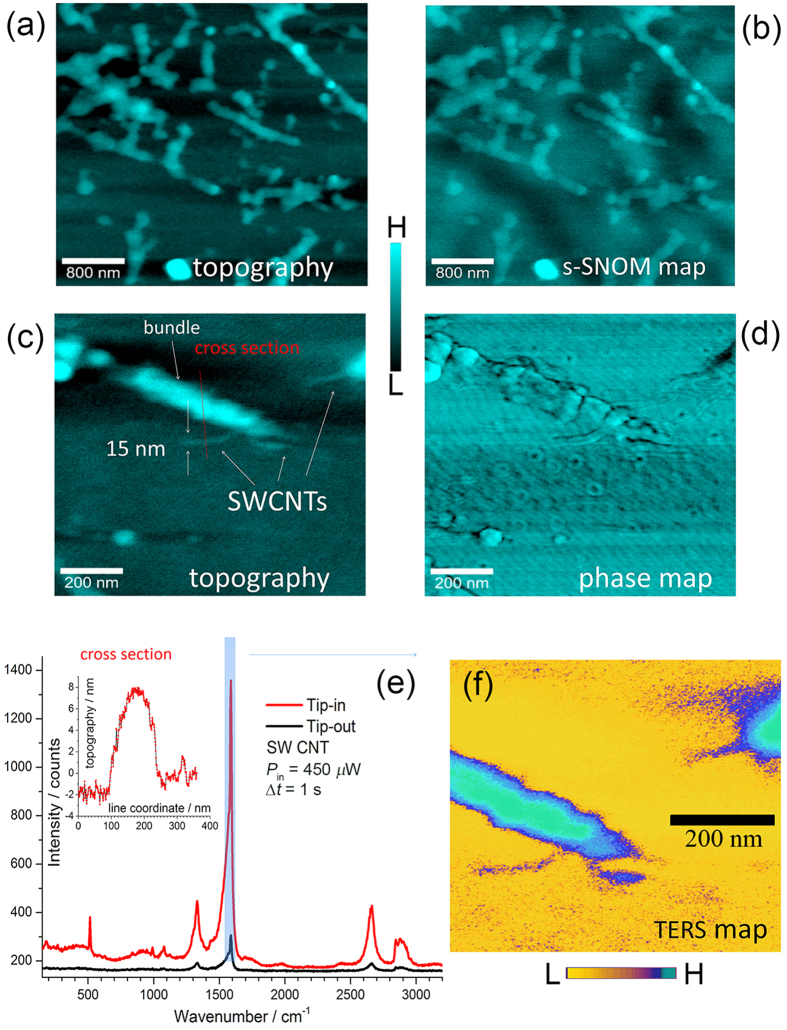
Experimental characterization of the TERS probes on SWCNTs. (**a**) Large area topography of bundles on SWCNTS spin-coated on glass and (**b**) corresponding s-SNOM map. (**c**) Topography of a magnified region of interest and (**d**) corresponding phase map; 512 × 512 pixels over an area of 1 *μ*m^2^. (**e**) Tip-in and tip-out signals onto a bundle of SWCNT; the inset shows the topographic cross section along the dashed line traced in panel (c). (**f**) TERS map onto a region of (**c**), acquired with incident power of 450 *μ*W, integration time Δ*t* = 15 ms per pixel.

Since the presence of near-field Raman photons implies also the detection of elastically backscattered Rayleigh photons, generally speaking, the opposite may be used to verify, preliminarily, the near-field origin of the detected photons. In other words, the absence of near-field Rayleigh signal correlated with the morphology of the scanned structure implies that TERS imaging is not achievable. Therefore, scattering-type SNOM map of SWCNT bundles were preliminarily acquired for checking out the near-field operation, as shown in Fig. 5(a,b). This had the advantage of carrying out very fast scans over large areas to identify the region of interest (see next section for further details). Then, TERS measurements were conducted on magnified areas.

The average enhancement factor *G*_av_ of the near-field probe can be written as


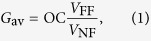


where OC = *I*_NF_/*I*_FF_ represents the optical contrast between the near field *I*_NF_ and far field intensities *I*_FF_, respectively corresponding to the tip-in and tip-out signals measured on a bundle of SWCNTs (band at 1588 cm^−1^). The far field volume of scattering *V*_FF_ is defined as the intersection of the sampling molecules volume and the confocal beam volume. The first was estimated from the AFM topographic map, from which an approximate volume of the SWCNT bundles can be extracted, whereas the second by a knife-edge Raman characterization^41^. The near field sampling volume *V*_NF_ was approximately estimated from the extent of the local field at the tip. Radially polarized excitation was preferred for these experiments as enabling both longitudinal and planar components on the tip due to the finite efficiency of polarization conversion of the liquid crystal plate (~40%).

In Fig. 5(c,d), respectively, the topographic and (simultaneous) phase maps of a region of interest are shown. In Fig. 5(e), the spectral signature of a SWCNT and the relative optical contrast under approached TERS tip is shown. The inset also shows a topographic cross section along a bundle of SWCNTs and an isolated SWCNT, with heights, respectively, of ~7 nm and ~1 nm. By setting *h*_NF_ = 7 nm, we have 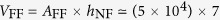 nm^3^. Estimating a tip-cluster radius of approximately 10 nm and an interaction length >7 nm, we obtain a conservative value of near field volume 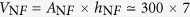 nm^3^. *G*_av_ is finally determined according to Eq. (1).

The average OC measured with this kind of characterizations was ~35 and corresponded to an approximate 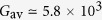. For the above mentioned measurements on SWCNT bundles, incident power was set at a constant level of 450 *μ*W with integration time Δ*t* = 1 s for both tip-in and tip-out measurements.

However, with same exposure conditions, spontaneous Raman was not detectable on isolated SWCNTs. Therefore we carried out another characterization on isolated SWCNTs with incident power set to 13 mW for spontaneous Raman detection (tip-out) and to 450 *μ*W for TERS (tip-in) (both with Δ*t* = 1 s). This gave a value 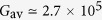. We ascribed the variability of these results to the larger absorption of the bundles with respect to the isolated SWCNTs, absorption that likely affected the intensity of the backscattering. Recalling that the supporting substrate was not metallic, we concluded that the above values of enhancement, in particular the results on isolated SWCNTs, were consistent with the simulated average enhancement factor at the outer surface of the apical clusters [Fig. 4(a)].

The spatial resolution was determined by imaging a SWCNT band at 1588 cm^−1^ and gave a value of ≃15 nm [Fig. 5(f)]. In this regard, we note that although the nanoisland structure has a size of the order of 30 nm, the actual TERS resolution is expected to depend mainly on the volume of the hot-spots in the *intra*- or *inter*-island gaps and their influence on the probed region.

#### BiA-TERS characterization

To further characterize the enhancement of the TERS probe, we used the same tip into a gap-mode TERS experiment using a planar SERS substrate made of the same nanostructure as bottom plasmonic mirror. Such SERS substrate is transparent, therefore allows gap-mode TERS operation in backscattering geometry. A 20 *μ*l-drop of aqueous solution of crystal violet (CV) and rhodamine 6G (R6G) (both 10 nM) was casted on the SERS substrate over an area of approximately 80 mm^2^ and left drying. Molecular surface densities were estimated to be of 1,500 molecules/*μ*m^2^ for each molecular species. Considering an occupied area of ≃1 nm^2^ per molecule, we estimated a total coverage ≃0.3% of the area of the substrate. We used this method to recreate a bi-analyte SERS experiment^60^ in a gap-mode TERS configuration (BiA-TERS). Preliminarily, the spatial reproducibility of the SERS amplification provided by the SERS substrate was accurately characterized giving a SERS intensity fluctuation <10%^41^. This permitted us to rule out major fluctuations in the detected gap-mode TERS signals due to the local character of the bottom substrate amplification.

**Figure 6:**
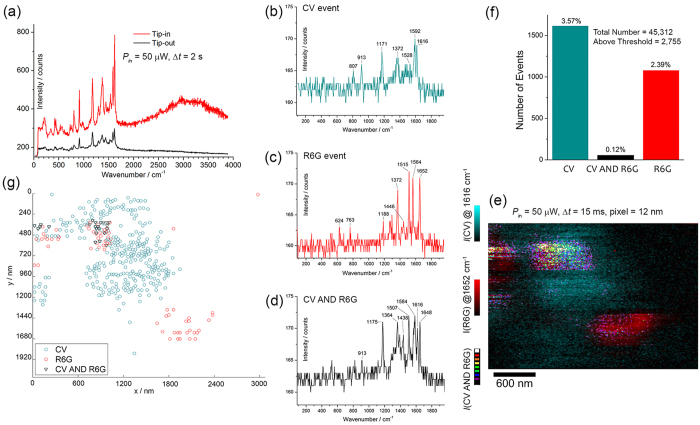
Bi-analyte TERS experiment. (**a**) Tip-in (TERS) and tip-out (SERS) spectra onto a transparent SERS substrate covered with CV and R6G molecules (with incident radial polarization). The SERS and gap-mode TERS spectra point out only the CV component into a fixed position. Panels (b–d) show examples of typical TERS spectra of CV, R6G and mixed CV with R6G (indicated as CV AND R6G) acquired in consecutive positions of the SERS substrate. (**e**) Normalized integrated intensity maps, respectively, of the main bands of CV (blue color) and R6G (red color) with overlaid colormaps and coincidence map of the product of the intensities of CV and R6G (dark level = 0, white level = 1). The map was taken with 256 × 170 pixels over an area of 3 *μ*m × 2 *μ*m, with integration time Δ*t* = 15 ms (per pixel). (**f**) Histogram of the counts of single molecule events of CV, R6G and mixed events. The low coincidence rate points out a single-molecule statistics. (**g**) Centroidal map of the regions of connected pixels in which one spectral species (CV, R6G or mixed) appear consecutively detected along the scan in (**f**).

The outcomes of the BiA-TERS experiment are shown in Fig. 6. In the first panel (a), we show the amplification produced when the TERS tip is in contact with the substrate, providing an additional gain of approximately 4 to the SERS amplification of the substrate. This further gain is a relative large enhancement since the gap area is estimated to be only ≃1/1000 of the substrate scattering area (0.44 *μ*m^2^). A straightforward calculation points out a multiplicative contribution of the gap-mode TERS configuration of 3 × 10^3^. Since the average enhancement factor of the SERS substrate was previously estimated to be 10^6^ with CV molecules^41^, we conclude that the TERS enhancement induced in gap-mode is of the order of 10^9^, a value consistent with those in principle achievable in the gap between tip and substrate.

At this point, an area of 3 × 2 *μ*m^2^ with a grid of 256 × 170 = 43,520 pixels of 12 nm of spacing was scanned for detecting the coincidence map of bi-analyte spectra. Figure 6(b–d) show gap-mode TERS spectra acquired in consecutive positions showing, respectively, pure events of CV, R6G and a mixed event of CV and R6G.

Figure 6(e) shows overlaid maps of the normalized intensities of the main TERS bands of CV and R6G combined with the product intensity map of the two molecules, indicated as CV AND R6G. On average, the TERS signals of R6G were relatively more intense into three defined regions. Inhomogeneous intensity maps could be caused by aggregation into water or molecular flows during its evaporation. The presence of a structured molecular distribution on the substrate pointed out a TERS signal correlated to the actual characteristics of the surface. This permitted us to rule out the possibility of false spatially random patterns produced, actually, by temporal fluctuations of the TERS signal during the scan, for example due to contamination of the tip.

Figure 6(f) reports the analysis of coincidence of TERS events of single CV and R6G and mixed molecules. Percentages indicated in the figure refer to the relative number of positive detections above threshold, normalized to the total number of scanned positions (Methods). As can be seen, the low rate of coincidence of CV and R6G is evidence of single-molecule statistics according to the bi-analyte SERS method. Figure 6(g) shows the map of centroids of the regions of pixels in which a continuous presence (connected pixels) of one particular spectral species, either CV, R6G or mixed event, was detected. We can observe a few defined regions of local concentration of molecules of CV and R6G with rare, very small regions of continuous pixels showing mixed molecular species. In fact, data analysis revealed that spectra acquired in consecutive positions mainly reported the signature of single analytes, either CV or R6G. Examples of spectra in consecutive positions are in Fig. 6(b,c). Therefore, this anlaysis indicates the capability of the nanostructure architecture on the tip to localize single (Raman resonant) molecules, in gap-mode TERS, with resolution at least of 12 nm.

#### TERS on a thick biosample: *B. subtilis* spore

**Figure 7:**
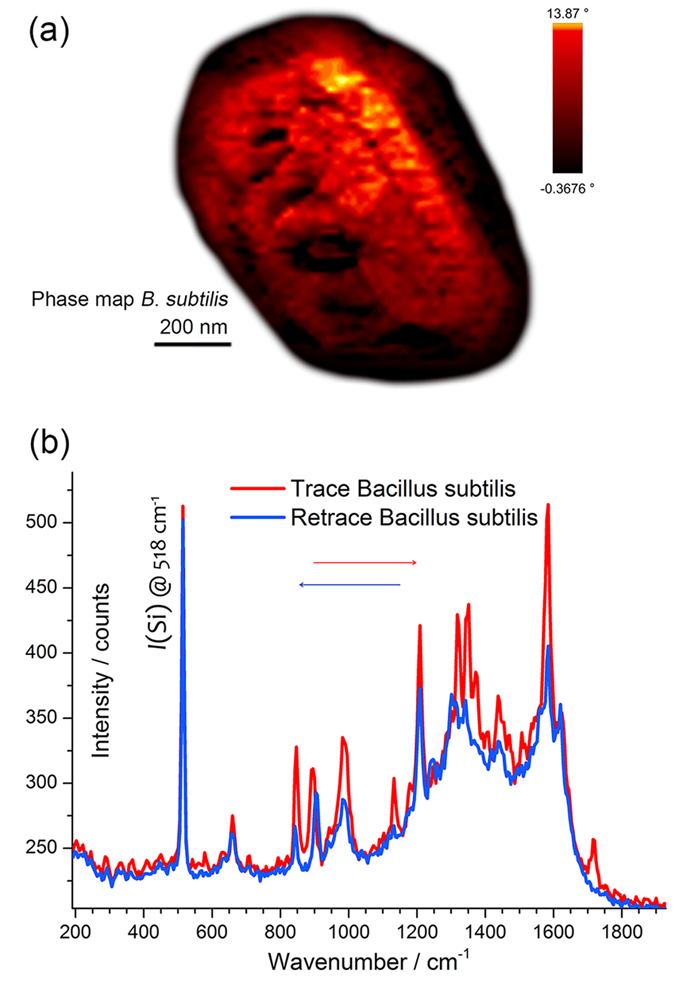
TERS characterization on a thick biosample: spore of *Bacillus subtilis*. (**a**) Phase map of *B. subtilis* spore. (**b**) Tip-in, trace and retrace TERS spectra onto the *B. subtilis* spore. See text for details.

Given the positive results obtained with our TERS probes on standard Raman analytes used to determine the characteristics of the tips, we then focused on a more challenging non-Raman resonant biosample. We applied the Arrow^©^ tip coated with Ag@AuNPs on a thick spore of wild-type strain of *Bacillus subtilis*^61^, also in this case in intermittent contact mode. Figure 7(a) shows the phase map of the spore. In this preliminary investigation, we limited the inspection to the detection of the outer coat chemical fingerprint, amplified by the TERS tip in point measurements or line scans, as shown in panel (b). It is important to observe, in fact, that the spore volume, with an height of ~1000 nm and a thickness of ~500 nm acts as a diffractive optical element in bottom illumination, therefore disturbing excitation of plasmonic tip. The detected TERS spectra were found in good agreement with those obtained in our previous investigations^40^,^61^. For not-engaged tip, no Raman bands were observed except for the silicon band at 518 cm^−1^ and a carbonaceous luminescence background, whereas, for tip engaged, several intense bands appeared in the spectrum, with good signal-to-noise ratio (Fig. 7b). With respect to ref. 40, we found an increased signal-to-noise ratio due to the larger scattering efficiency of the probe. In the present case, we confirmed the near field origin of the TERS peaks by tracking the TERS spectra during trace and retrace positioning along a line scan on the top of the spore. Overall, a good matching between these spectra was observed, an example of which is shown in Fig. 7(b).

### Experimental Application: s-SNOM

**Figure 8:**
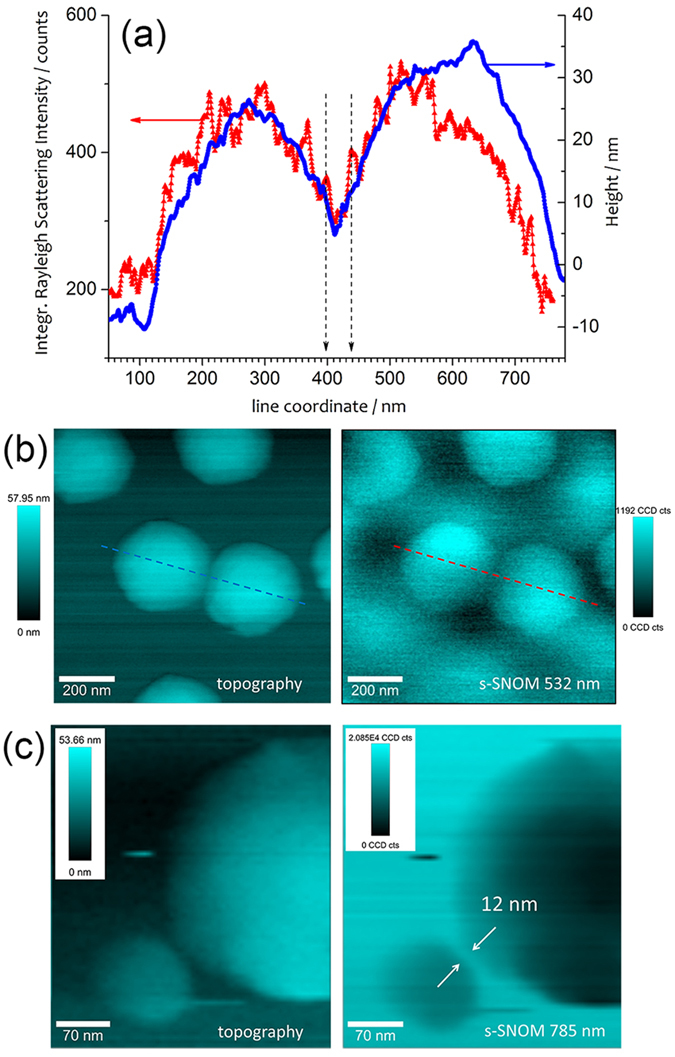
s-SNOM measurements on a patterned gold nanostructure. (**a**) Topography and integrated Rayleigh intensity (s-SNOM) along the cross section indicated in (**b**), acquired in contact mode with a Ag@AuNP coated tip, with incident power of 100 nW at 532 nm and integration time of 15 ms per pixel, with incident radial polarization, over an area of 1 *μ*m^2^ (256 × 256 pixels). (**c**) Detail of closely spaced gold nanopillars (topography and s-SNOM), extracted from a scan area of 350 × 350 pixels over 4 *μ*m^2^ (step of 5.7 nm), measured with excitation laser power of 150 nW at 785 nm, with radial polarization and integration time of 12 ms per pixel. All colormaps represent signal amplitudes leveled to the minimum.

In order to provide an independent characterization of the spatial resolution of the coated probes, we applied an Ag@AuNP-coated Arrow tip in contact mode for s-SNOM experiments on gold nanopillars fabricated on ITO-coated glass with electron beam lithography^62^. In particular, we used either 532 or 785 nm as excitation wavelengths, with the same optical configuration used for TERS measurements. Figure 8(a) shows both topographic and s-SNOM cross sections acquired across the gap of two close pillars. s-SNOM signal is obtained by integrating the intensity of the elastic Rayleigh backscattered light from the tip (notch filter removed). In Fig. 8(b,c), we show the s-SNOM maps that were obtained on two regions of interest of the nanostructure, characterized by 10-nm gaps between two gold pillars (right panels). Simultaneously acquired topographic maps are shown for comparison and demonstrate an excellent spatial resolution (left panels). Incident power on the sample was limited to 150 nW, with integration time of 15 ms. Although faster scanning was not possible because limited by the electronics of our setup, we found s-SNOM mapping to be possible even with incident power <10 nW, therefore indicating the possibility to reduce integration time <1 ms. No phase detection scheme was adopted for technical limitation of our optical setup. However, the presence of a background field did not prevent the clear identification of the nanostrucures, likely because of the large signal-to-noise ratio provided by the intense scattering from the tip at the laser wavelengths used. As shown in Fig. 8(c), in which a magnified s-SNOM map acquired at 785 nm is reported, besides the large optical contrast, an excellent spatial resolution was found and estimated to be <12 nm.

The possibility to use the BCP-coated probes also for s-SNOM is directly related to their large scattering efficiency that allows facile nongap-mode operation, as also shown in Fig. 5 on CNTs spin-coated on glass.

## Discussion

This study demonstrates a versatile probe functionalization based on BCP/metal nanocomposites for nanospectroscopy applications. Our approach enables producing highly dense clusters of nanoparticles with controlled size dispersion since NPs are nucleated within BCP micelles with overall size commensurate with the tip apex. BCP self-assembling produces disconnected nanoislands with nanometric gaps. In principle, the unit structure of the hierarchical assembly may be also optimized to address specific requirements before being conveyed to the tip by self-assembling. Of course, the possibility of coating complex 3*D* structures like a challenging AFM tip is a great advantage that can found many further applications.

The large local field amplification at the gap-hot spot in the dimer tip-mirror is the basis of gap-mode TERS. A tip coated with clusters of NPs is intended to promote a cascade amplification in sub-nanometer gaps at the apical clusters on the tip (Fig. 4). Our numerical simulations indicate the possibility of large amplification on the outer surface of the cluster (10^5^–10^6^). This is confirmed experimentally on SWCNTs without a bottom plasmonic mirror (Fig. 5). This last is an important advantage that provides access to the surface chemistry of thick samples and is demonstrated onto a *B. subtilis* spore with good signal-to-noise ratio (Fig. 7). Moreover, the ultrasensitive detection verified with the bi-analyte gap-mode TERS experiment in Fig. 6 points out the possibility to achieve a very large enhancement factor in the gap between the nanoisland at the tip and a bottom SERS nanostructure. With respect to previous works on single-molecule TERS^63^,^64^, the innovation of our approach relies on the use of a nanoscale, spatially resolved version of the bi-analyte method that allows us establishing the onset of single-molecule regime taking also into account experimental issues due to the extreme-value statistics of the enhancement factor at the gap hot-spot^60^.

The fine grain of the nanostructure showed an excellent spatial resolution (<12 nm). This was verified in bi-analyte TERS and confirmed in s-SNOM experiments on a gold nanostructure.

While for a dimer the local field amplification depends dramatically on the particular alignment with respect to the incident polarization, in our case, the formation of BCP-nucleated NPs is expected to provide a more isotropic electromagnetic response with respect to the incident polarization. In addition, the cluster broadens the optical response of the system, which in turn increases the probability to match the frequency of a localized surface plasmon resonance. Of course, the actual efficiency of our functionalized probes, since based on a fractal, random geometry of NPs at the apex, is subject to the particular geometry of the nanostructured tip. One of the advantages introduced by nucleation of NPs into BCP micelles relies on the possibility of reducing the dispersion of the cluster nanostructure. We found an overall good rate of reproducibility with 7 tips with AgNP-coating giving large enhancements in a batch of 10. The outcomes with AuNPs were more variable. However, much research can still be done to improve these results. So far, we did not find remarkable differences between Ag-coated tips and mixed Ag and AuNPs (1:0.7) in terms of TERS efficiency. Both had comparable enhancements, larger than what was found in the other cases here examined. A discriminant factor between them was found in the different chemical interaction of the probes with the biosample of *B. subtilis*. So far, fluctuations of the signal were larger in the case of AgNPs. A more systematic investigation is still in progress.

Residual contamination of carbonaceous species produced from irradiation of copolymer is an important drawback of our approach. However, post-delivery treatments like thermal annealing or low energy oxygen/hydrogen plasma etching may be investigated to remove the organic contamination more efficiently. Further research should be also conducted to explore different scattering geometries.

The relatively time-consuming synthesis procedure may be also a limitation. However, the shelf time of the solution can be of many weeks. In addition, only 20 ml of final solution, in principle, are required to coat more than 200 tips.

In perspective, introducing a synergistic chemical functionalization of the tips might address specific molecular targets, allow novel detection mechanisms (like plasmonic response activation) or endow the near-field probes with a response targeting the material under investigation by virtue of specific nanocrystals coating. In fact, micelles may convey to the tip other kinds of nanocrystals^38^ loaded by protonation (Pt, Co, CoPt, Fe2O3, FePt, ZnO, TiO2, SiO2 etc.), which might expand the capability of functionalization of the probes.

Combining a reproducible synthesis approach for versatile inclusion and delivery of plasmonic nanoparticles at the tip apex, which may promote the tuning of the plasmonic properties, a large enhancement, and the possibility of adding new degrees of freedom for tip functionalization, is promising for engineered applications of nanospectrospies.

## Methods

### Optical setup

All experiments were conducted with a combined AFM and confocal Raman microscopy system WITec alpha 300 with bottom illumination and backscattering collection. Illumination of the tip was accomplished with a radially polarized beam produced by a liquid crystal plate (ARCoptix, Switzerland). The confocal system was coupled with a spectrometer equipped with two diffraction gratings with 600 and 1,800 g/mm providing a resolution, respectively, of 3.6 and 1.5 cm^−1^. Backscattering light collection and detection were, respectively, through a Nikon 60× dry objective (NA = 0.8 and WD = 300 *μ*m) and a deep-depletion, back-illuminated Andor CCD camera (DV401A-BV-352) (1,024 × 128 pixels) operating at −60 °C. The confocal condition was imposed by the core (acting as a pin-hole) (25 *μ*m) of the multimode fiber delivering the signal to the spectrometer. The backscattering area on the sample *A*_scat_ was accurately measured independently to be 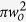 = 0.44 *μ*m^2^ (beam waist *w*_*o*_ = 373 ± 3 nm) with a knife-edge technique using the Raman intensity of a silicon wafer. A three-axis piezo-positioner allowed precise control of the sampling translation with nanometer accuracy over a maximum range of 100 × 100 *μ*m^2^.

### BCP-metal nanocomposite synthesis and deposition

Polystyrene-*block*-poly-4-vinylpyridine with number-average molecular mass of 10,400-*b*-19,200 g/mol was purchased from Polymer Source Inc. and used as is. All chemicals for NP synthesis were purchased from Sigma Aldrich. A mixture of tetrahydrofuran (THF) and toluene (ratio 0.67 w/w) was used to form BCP micelles. 97.2 mg of PS-*b*-P4VP were added to 20 ml of solvent, *i.e. c* = 0.55% w/w. The solution was stirred at 700 rpm for 3 h at 25 °C and then for 2 h at 67 °C. Further details can be found in ref. 41. Neat micelles solutions were characterized by dynamic light scattering and small angle X-ray scattering. A value of polydispersity of ca. 8% was initially measured.

BCP micelles were loaded by complexing the P4VP core either with Ag^+^, from 203.7 mg of AgNO_3_ in 20 ml of solvent, or with Au^3+^, from 470 mg of HAuCl_4_ ⋅ 3H_2_O, both corresponding to a concentration of 0.55% w/w of BCP in solution to produce fully metal core of AgNP clusters and AuNP clusters (this only for additional tests), respectively. Solutions were purified to remove supernatant micelles after centrifugation at 15 krpm for 1 h. NaBH_4_ was used to reduce the core-loaded ions to metal nanoparticles. Ag-loaded BCP solution was left under stirring in dark ambient condition for two weeks in order to induce metal seed overgrowth into the micelle core. We estimated the fraction of AgNO_3_ dissolved in the solution and mainly contributing to the micelle’s cargo by weighing unreacted salt. The unreacted fraction was about 75% (~150 mg). At this point, several amounts of HAuCl_4_ ⋅ 3H_2_O were added to the Ag-loaded BCP solution, corresponding approximately to molar ratios with dissolved fraction of silver nitrate given by Ag:Au = 1:0.3, 1:0.6, 1:1.1. AuNPs were produced by reduction with NaBH_4_ already present in solution, then newly nucleated NPs were found attached to the BCP micelles (Fig. 1b). The molar ratio ratios were found in good agreement with relative atomic concentration of Ag and Au determined from EDX spectroscopy (Fig. S1 in SI) performed on film deposited on silicon wafers, and used as reference in Fig. 2, *i.e.* Ag:Au = 1:0.3, 1:0.7, and 1:1.2. Then, a 2-ml solution (whether before or after AuNP formation) was centrifuged at 11 krpm for 20 min to purify the solution employed for dip coating by drawing desired amounts of liquid (typically 10^2^ *μ*l) at a height corresponding to heavier loaded micelles.

Typically, 100-*μ*l solutions were spin-coated over commercial glass coverslips at 1.0-krpm speed for 60 s to form planar SERS substrates. The solutions were filtered before deposition on glass coverslips with 200 nm PTFE syringe filters. UV-vis spectra were acquired with Perkin Elemer Lambda 35 equipped with integrating sphere. TERS tips were instead coated by dip-coating as described in the text. UV irradiation at 254 nm from a commercial Hg lamp in air for a period of 24 h allowed to remove the polymer.

SEM images were obtained with a field emission SEM FEI Nova NanoSEM 450 at an accelerating voltage of 2 kV (range of acceleration voltage: 50 V to 30 kV) equipped with a Through Lens Detector and a Zeiss Supra 40VP FE-SEM with In Lens Detector.

Thin films of BCP nanocomposites for TEM analysis were backed with a carbon film, floated off on water with the aid of a poly(acrylic acid) backing, mounted on copper grids and analyzed by a Philips EM 208S microscope operating at a voltage of 120 kV (point resolution of 0.3 nm).

### BiA-TERS

Preliminary spatial reproducibility of the SERS substrate was characterized using a uniform monolayer of CV molecules deposited on the substrate, according to our previous methods^41^. For bi-analyte statistics measurements, acquired TERS spectra were deconvolved along the two spectral components of CV and R6G by using the basis analysis described in refs 40 and 41 Positions of positive detection (or null detection for insufficient signal level) were defined by imposing a threshold value, equal to fivefold the noise amplitude, on the background-subtracted intensity of the Raman bands. SERS spectra of single-molecule events were characterized by spectral wandering. In particular, the bands centered at 1620 cm^−1^ for CV and 610 cm^−1^ for R6G showed a spectral fluctuation of Δ*ν* = |1616 − 1622| = 6 cm^−1^ and Δν = |623 − 614| = 9 cm^−1^, respectively. The SERS substrate had alone an enhancement factor ≃10^6^ for radially polarized excitation. A further gain of 4 with the tip engaged over a contact area of only ≃1/1000 of the total scattering area of 0.44 *μ*m^2^ indicated a gap-enhancement 3000 larger, *i.e.* of the order of ~10^9^.

### Numerical simulations

Numerical simulations were carried out with a commercial software based on the finite element method. The spherical simulation region was embedded into a perfectly matched layer with 10 shell elements to avoid backreflections. In addition, outer surface elements satisfied scattering boundary conditions according to the propagation direction of the incident excitation. Minimum element size in mesh calculation was 0.07 nm. The simulations were carried out over a range of wavelengths spanning from 200 to 750 nm, with step size of 2.5–5 nm. Stability of simulations were verified against mesh size. Relative error tolerance was set to 1 × 10^−7^. The surface charge density on the NPs was calculated by the relation 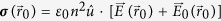, where 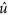 is the normal versor to the surface of the nanoparticles, *n* the refractive index of the surrounding medium, 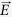 is the scattered field, 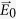 the background field and 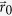 indicates the position on the NP surface.

## Additional Information

**How to cite this article**: Zito, G. *et al*. Nanometal Skin of Plasmonic Heterostructures for Highly Efficient Near-Field Scattering Probes. *Sci. Rep.* **6**, 31113; doi: 10.1038/srep31113 (2016).

## Supplementary Material

Supplementary Information
