# Supplementary material for: Nanometal Skin of Plasmonic Heterostructures for Highly Efficient Near-Field Scattering Probes
Source: Sci Rep. 2016 Aug 9;6:31113. doi: 10.1038/srep31113 (PMC4977468; doi:10.1038/srep31113)
Supplement: Supplementary Information [file srep31113-s1.pdf]

Supplementary Information

Nanometal Skin of Plasmonic Heterostructures for Highly Efficient Near-Field Scattering Probes

Gianluigi Zito,[*,†] Giulia Rusciano,[‡] Antonio Vecchione,[¶] Giuseppe Pesce,[‡] Rocco Di Girolamo,[§] Anna Malafronte,[§] and Antonio Sasso[‡]

Department of Physics E. Pancini, University of Naples Federico II, via Cintia, 80126-I Naples, Italy, Department of Physics E. Pancini, University of Naples Federico II, via Cintia, 80126-I Naples, Italy., CNR SPIN U.O.S. and Department of Physics, University of Salerno, via Giovanni Paolo II 132, 84084 Fisciano (Sa), Italy., and Department of Chemical Sciences, University of Naples Federico II, via Cintia, 80126-I Naples, Italy.

E-mail: zito@fisica.unina.it

[*]To whom correspondence should be addressed

[†]Department of Physics, University of Napoli Federico II

[‡]Department of Physics E. Pancini, University of Naples Federico II, via Cintia, 80126-I Naples, Italy.

[¶]CNR SPIN U.O.S. and Department of Physics, University of Salerno, via Giovanni Paolo II 132, 84084 Fisciano (Sa), Italy.

[§]Department of Chemical Sciences, University of Naples Federico II, via Cintia, 80126-I Naples, Italy.

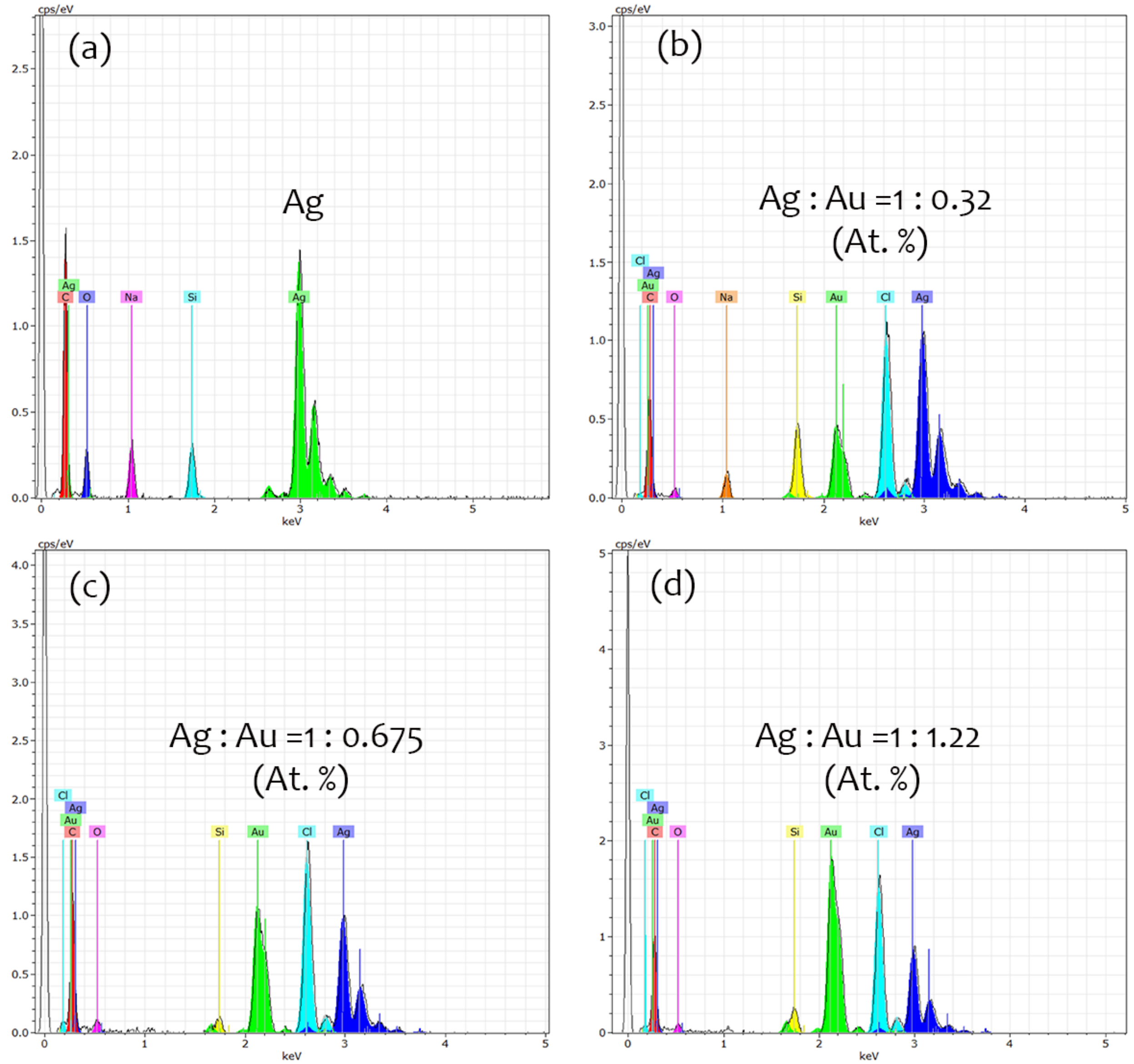

Figure S1: (a) Energy dispersive X-ray (EDX) spectrum of a film obtained from BCP micelles loaded with AgNPs. (b) EDX spectrum from BCP micelles loaded with AgNPs, then decorated with AuNPs in molar ratio Ag : Au = 1 : 0.31, which is in good agreement with the atomic ratio determined from deconvolution of EDX peaks, equal to 1 : 0.32. (c) EDX spectrum from BCP micelles loaded with AgNPs, then decorated with AuNPs in molar ratio Ag : Au = 1 : 0.60, in good agreement with the EDX atomic ratio equal to 1 : 0.675. (d) EDX spectrum from BCP micelles loaded with AgNPs, decorated with AuNPs in molar ratio Ag : Au = 1 : 1.08, in good agreement with the EDX atomic ratio equal to 1 : 1.22. For all samples, a silicon wafer was used as supporting substrate.

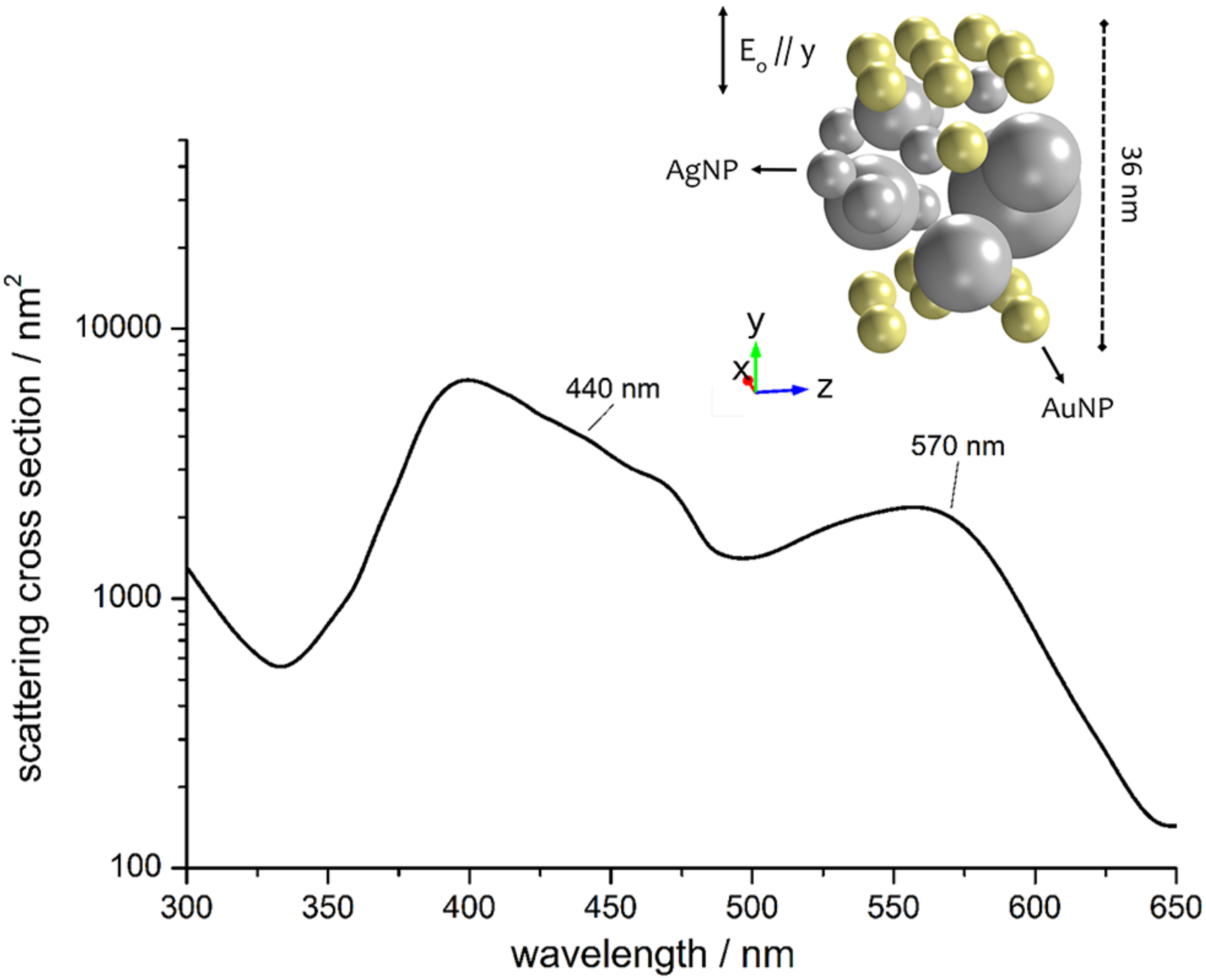

Figure S2: Scattering cross section spectrum calculated for the cluster geometry depicted in the inset and consisting of mixed Ag- and Au-NPs, for plane wave excitation with vertical polarization. The far-field optical response here highlighted is in good *qualitative* agreement with the experimental optical response, in particular, in relation to the emergence of a lower energy band (550-570 nm) due to hybridization of AgNPs and AuNPs response. No relevant variation is found in the case on Ag- and Au-NPs mixed with more heterogeneity. The geometry of the cluster was tentatively extrapolated from the various morphological characterizations. All AgNPs were $<$ 15 nm and AuNPs had diameters of 5 nm.
